# A ‘Suitable Soil’: Plague’s Urban Breeding Grounds at the Dawn of the Third Pandemic

**DOI:** 10.1017/mdh.2017.32

**Published:** 2017-07

**Authors:** Christos Lynteris

**Affiliations:** University of Cambridge, CRASSH, Alison Richard Building, 7 West Road, Cambridge CB3 9DT, UK

**Keywords:** Plague, Hong Kong, India, Soil, Experiments, Colonial medicine

## Abstract

A pressing question during the first half-decade of the third plague pandemic (1894–9) was what was a ‘suitable soil’ for the disease. The question related to plague’s perceived ability to disappear from a given city only to reappear at some future point; a phenomenon that became central to scientific investigations of the disease. However, rather than this simply having a metaphorical meaning, the debate around plague’s ‘suitable soil’ actually concerned the material reality of the soil itself. The prevalence of plague in the working-class neighbourhood of Taipingshan during the first major outbreak of the pandemic, in 1894 in Hong Kong, led to an extensive debate regarding the ability of the soil to harbour and even spread the disease. Involving experiments, which were seen as able to procure evidence for or against the demolition or even torching of the area, scientific and administrative concerns over the soil rendered it an unstable yet highly productive epistemic thing. The spread of plague to India further fuelled concerns over the ability of the soil to act as the medium of the disease’s so-called true recrudescence. Besides high-profile scientific debates, hands-on experiments on purifying the soil of infected houses by means of highly intrusive methods allowed scientists and administrators to act upon and further solidify plague’s supposed invisibility in the urban terrain. Rather than being a short-lived, moribund object of epidemiological concern, this paper will demonstrate that the soil played a crucial role in the development of plague as a scientifically knowable and actionable category for modern medicine.

‘Every medical man will tell you that plague has never been stamped out. It has always appeared again some year later’.^1^ The statement of Dr Boucher before the Mauritius Special Committee on Plague in 1899 reflects a key scientific and administrative concern regarding plague at the turn of the nineteenth century: its periodic reappearance in specific localities. Following the first major outbreak of the third plague pandemic and the discovery of the plague bacillus in Hong Kong in 1894, this question largely revolved around where the disease lay hidden when absent in human populations. Whilst entangled with the question of origins, the quest for the so-called breeding grounds of plague assumed an epistemologically autonomous status that was closely tied to understandings of the particular disease as an urban phenomenon. This question was not simply focused on filth as an epistemic conveyor between bacteriological and non-bacteriological understandings of the disease. Instead it constituted a new object of scientific knowledge relating to the study and control of plague: the soil.

In what, following David Barnes, we may call a ‘sanitary-bacteriological synthesis’ characteristic of late nineteenth-century medicine, during the first years of the third plague pandemic, the soil came under multilayered medical suspicion and scrutiny.^2^ This no longer related to ideas of plague being caused by gases emanating from the earth, but instead to the notion that the soil was a physical carrier and spreader of the disease.^3^ On one level, the soil was seen as a potential source of human infection through direct contact and inoculation, for example through walking barefoot. On a second level, it was seen as a reservoir where plague concealed itself during shorter or longer periods of latency. In the second case, though the soil was not seen as directly infectious, it was considered as the proper medium of plague; in other words, the habitat of plague’s so-called true recrudescence. In the first half-decade of the pandemic (1894–9), the two hypotheses formed the basis for extensive research and epidemic control practices and deliberations. The configuration of the soil as the breeding ground of plague was rooted in bacteriological methods of rendering infectious disease knowable. And, at the same time, it was grounded in colonial epidemic control policies that often relied on sanitary frameworks of public health. Rather than, however, simply providing a temporary compromise between bacteriology and sanitarianism, the soil’s efficacy in terms of epidemiological reasoning relied on its indeterminate character as a scientifically knowable and actionable urban materiality.

Precariously forged between the laboratory and the street, the question of the soil came to concern not only the matter that composed it, but also whatever was erected or lived on it. Questions about the relation of plague and the soil hence assumed critical implications for the structures built on the soil, as well as for the ways of life unfolding on its surface. In this sense, when it came to its configuration as the breeding ground of plague, the soil became the ground for the emergence and transformation of medical ideas regarding the role and interrelation of housing, planning and native custom in the generation and spread of the disease in the colonial city.

Recent scientific studies have fuelled new interest in the possibility of the soil being in fact implicated in the persistence or spread of plague.^4^ Yet, unlike Mollaret’s 1963 classic study, this paper is not a historical review of this research.^5^ In contrast to Mollaret, who was driven by the conviction of her mentor (the Pasteurian Girard) that this was ‘the one and true unsolved problem connected with plague’, my interest here lies in the way in which, at the beginning of the third plague pandemic, research on the role of the soil implicated and entangled urban forms and lifestyles in the aetiology of plague.^6^ My aim then is to demonstrate how the question of the soil impacted on a series of debates that preoccupied doctors and administrators at the advent of the third pandemic: what environments were conducive to the rise and spread of plague? What urban structures harboured the bacillus? What housing forms gave refuge to the disease? And, at the same time, what native lifestyles allowed plague to spread or persist in urban environments?

By examining the epidemiological trajectory of the soil as a carrier and spreader of plague in colonial Hong Kong and British India at the turn of the century, this paper underlines the need to ground both historical understandings of the development of medical ideas and debates regarding plague-conducive urban structures and behavioural patterns on a re-evaluation of the soil as a short-lived but catalytic object of plague science.

## 1 Soil Experiments in Hong Kong

I am of the opinion that the disease was introduced here from Canton, and, finding a suitable soil for development, it multiplied here.

Sir William Robinson to the Marquess of Ripon, 17 May 1894^7^

Soon after plague arrived in Hong Kong in May 1894, British colonial authorities noticed that the working-class, predominantly ‘coolie’ area of Taipingshan (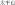) accounted for the majority of plague cases. This led doctors and administrators to raise questions about the disease, primarily not in terms of its ontology but in terms of its locality.^8^ Before the discovery of the plague bacterium (14 June 1894 by Kitasato and 20 June 1894 by Yersin), prevalent ideas about the cause of the disease revolved around it being some sort of miasma or telluric gas.^9^ James A. Lowson, acting superintendent of the Government Civil Hospital who was put in charge of the plague crisis, argued, ‘that poison is probably developed from atmospheric conditions underneath houses in certain districts, and that it is caused by poverty and dirt’.^10^ Lowson maintained that, ‘in the ordinary sense the disease is not infectious or contagious’, yet spending time together with an infected individual ‘in the same atmosphere’ could lead to contagion. The reason for this was that, ‘a very large concentration of patients in one place might lead to a concentration of poison and smell’.^11^ Though part of a wider Victorian nosological imaginary, this atmospheric theory sat uncomfortably with recent debates about the origin and mode of transmission of plague. In the course of mid-nineteenth-century epidemics in Mesopotamia, Persia and the Volga region, leading experts had systematically challenged ideas about plague emanating from the earth, suggesting various alternatives, including eating the meat of infected camels.^12^

Nonetheless, Hong Kong medical authorities sought to validate their opinion through a creative reading of ethnographic data regarding the perception of the disease in South China, where it was believed to have been raging for several decades. Stemming from the increased prevalence of the Warm-Factor School in Chinese medicine at the end of the nineteenth century, ideas about disease issuing from the earth quickly assumed popularity in the region. Carol Benedict has explained how plague was believed to be caused by pestilent earth *qi* (not sky *qi*). It was hence seen as a disorder that first affected rats; the heat of the earthly *qi* drove them out, where for relief they drank from unguarded cisterns or teacups, from whence humans received the pestilent *qi*.^13^ In the writings of British and French doctors operating in South China, this was translated into the idea that, as plague arose from the earth, it first infected animals which lived closer to the ground, such as rats, exponentially reaching taller creatures.^14^ Reproduced in official reports as well as in the daily press, it was a theory equally entangled with Western medical notions regarding objects as carriers of disease.^15^ Although it did not exclude ideas about disease self-generation, this outbreak narrative was primarily linked to a persisting category of epidemiological suspicion: filth.^16^ As Robert Peckham has shown, the prevailing notion that Taipingshan houses were nests of accumulated filth led to extensive operations, headed by the Shropshire Regiment, to remove and burn the contents of these houses out in the street, so as ‘to prevent contagion from the deposit of foul exhalations on their surface’.^17^ Ideas about objects being able to first absorb and then release pathogenic miasma were not new.^18^ What made the Hong Kong case distinct was how these were linked to particular concerns about things and spaces Chinese – a fear that eventually configured coolie houses upon the images of the opium den, aptly described by Peckham as a pathological topos: ‘here human beings are reduced to “forms” and objects are rubbish, a place where, as Lowson notes, “delirium dissipates coherency” ’.^19^ This all-encompassing pathologisation of what we may call coolie urban life was concurrent with growing colonial concerns about the most massive and well-organised coolie strike experienced in the colony for a decade.^20^ A paper in the *British Medical Journal*, covering Governor Robinson’s retrospective evaluation of the outbreak, underlines the class aspect of the colonial outbreak narrative:

By this time it is fairly established that plague is a filth disease, and that the habitations of the poorer classes of Chinese present for its purpose the most fertile soil in the world. The word ‘soil’ comes, not only figuratively, but actually, near the mark, for a Chinaman of the coolie class seldom or never cleans his abode, but allows the refuse of his household to litter the floor, where it gets packed and trodden down into a veritable midden.^21^

Yet the supposed filth of coolies, to whom ‘cleanliness […] or anything approaching it, is an absolutely unknown art’, was not the only topos of plague-related knowledge and intervention in Taipingshan.^22^ Drawing on Osbert Chadwick’s general sanitary survey of Hong Kong in 1882, concerns about the neighbourhood’s sewage systems led to the idea that ‘the soil of Taipingshan was typically soaked with sewage discharged from dysfunctional drains and through the broken floors of the buildings above’.^23^ Sewage was believed to ‘saturate’ the soil ‘in and around Chinese dwellings’, because their floors were made of ‘natural earth, or porous tiles’.^24^ Colonial disdain for such infrastructural defects, ‘permitting the percolation of sewage into the soil’, was matched with an equally dismissive attitude towards Chinese uses of proper sewage systems: ‘the Chinese are so ignorant and careless in all matters of drainage that the new methods of drainage, sound and good in themselves, are so abused that the effect is very little less injurious than that of the old method’.^25^ ‘Until this is remedied’, argued the chairman of the Sanitary Board, J. Francis, ‘there is no possible preventative against Plague, Typhus, Cholera and other diseases of the same character, nothing to prevent plague from becoming endemic in Hong Kong’.^26^ The solution proposed was the removal of all drainage and drainage openings from underneath Chinese house floors, and the replacement of existing infrastructures with ‘an impervious ground floor in and around every Chinese house’.^27^ Far from a simple process of retrofitting, this involved radical urban reform, as it necessitated, in the eyes of the Sanitary Board, the prohibition of back-to-back housing, which at the time comprised the prevailing urban form in the area.

Equally prevalent were concerns that the soil under the improperly built houses in Taipingshan might become the receptacle of plague bacteria ‘falling’ from infected items or bodies through the porous or gap-ridden floors of coolie abodes. In the words of Surgeon-Major James, this posed a central problem for the sanitary resumption of the area: ‘That the imperfect floors and the probable contamination of the soil by plague germs would be in all probability the means of rendering any new houses built upon the undisturbed soil unhealthy and possibly the starting point of a new outbreak’.^28^ Hence, rather than being a mere metaphor, a relic of miasmatic theory, or an aspect of the broader question of filth, as Mary Sutphen seems to suggest, in the course of the late stages of the Hong Kong plague outbreak (July–August 1894) the soil rose to an autonomous epistemic position as regards the spread and persistence of the disease.^29^

On 13 July 1894, The Committee for Housing the Chinese, the institution at the centre of the debate regarding the fate of Taipingshan, commenced a formal process of procuring evidence about the necessity of destroying designated houses. Should these be demolished or simply disinfected and re-populated? Should demolition be limited to houses which needed to be taken down in order to widen streets, and allow for more air and sunlight? Or should entire blocks be put to the torch? Should the soil in the area be disinfected, and was it safe to dig it and transport it to another location? The formal examination of evidence revolved around the opinion of the Colonial Surgeon, Dr Ayres, that the entire neighbourhood should be ‘destroyed, as far as possible, by fire’.^30^ Ayres indicted the soil as being both the source and medium of infection:

I say that the sub-soil is strongly infected and cannot be thoroughly dealt with as long as the buildings stand over it. If it were not so, you would not see rats coming out of sewers and holes in the ground, full of bacteria, not communicated to them from human patients but from the sewage which is soaking through the soil, and from the gases down below, a sure proof that the sub-soil is in a dangerous state. The rats do not catch the disease from any infection above, but from below.^31^

Proposing complete demolition, followed by digging up and disinfecting the soil, Ayres’s thesis raised concerns over the possibility of the digging or stirring up the soil causing further infection, a phenomenon allegedly observed during a ‘bilious remittent’ fever epidemic in Mauritius. Could burning be a solution to this problem? Though doctors like Major-Surgeon Preston believed that the products of the fire (ashes, charcoal, etc.) would have ‘a good effect’ on the soil, doubts were raised over the disinfecting potency of heat itself.^32^ Ayres objected by suggesting that dropping all timber onto the ground would create a fire that would burn for days, hence allowing sufficient heat to penetrate and purify the soil. As made explicit by the Hong Kong Director of Public Works, Francis A. Cooper, in a later stage of the Taipingshan resumption debate, this idea reflected the widespread belief that the London Fire of 1666, coming a year after the great plague outbreak, put an end to the disease in the city.^33^

Still not everyone agreed with this reading of evidence. Under examination, Dr Hartigan objected that only some houses needed to be pulled down; in others, replacing porous tiles with properly insulating floors would provide sufficient sanitary protection. If Ayres insisted that ‘the sub-soil is poisonous all over’ and the only solution was to ‘destroy the whole lot’, Hartigan cool-headedly objected that no studies had yet proved the existence of the bacillus in the soil. Still Ayres’ opinion of total demolition was seconded by influential medical figures in the colony. T.H. Knott, of the Royal Naval Hospital, stated that saturating the earth to a depth of three or four inches with carbolic acid would allay ‘fear of a recrudescence of disease from any germs that may be lurking in the soil’.^34^

The debate was not limited to the chambers of the colonial government. As shown by Cecilia Chu, the daily press reflected the opposing opinions, with Granville Sharp, a leading philanthropist, throwing his weight behind the milder approach.^35^ In order to elucidate the question, the Committee sought the advice of the two men claiming to have discovered the plague bacillus: Kitasato Shibasaburō and Alexandre Yersin. Sutphen has speculated that what first drew Kitasato to the examination of the soil as a potential carrier of plague was his training under Robert Koch.^36^ Be this as it may, in a manner consistent with his well-recorded patronage of Kitasato, James Lowson decided to procure the doctor’s opinions by taking a walk together in plague-stricken Taipingshan.^37^ During their urban promenade Lowson asked Kitasato what he thought about soil infection. The latter reportedly answered ambivalently, saying that he had been able to isolate a plague bacillus only once ‘in the dust of a house’.^38^ More experiments would be needed to decide one way or another. In the meantime, Kitasato recommended the removal of soil from houses ‘with earthen floors where the soil had been polluted with filth to an incredible extent’.^39^ In his reply to Ayres’ letter inviting his expert opinion on ‘questions regarding soil’, he added: ‘if the infected dwellings are to be totally purified and freed from any plague germs a radical treatment ought to be enforced, the best is to burn completely at least the inner parts of the houses’.^40^

In spite of his propensity for *plein air* bacteriology, Alexandre Yersin was not taken for a convivial walk.^41^ Still, he too was invited by Ayres to submit a written report on his findings regarding plague and the soil. The Pasteurian eagerly assumed the task, which besides giving him the opportunity to investigate plague in situ was also a solid, if belated, token of recognition of his scientific authority by the British. In his fourth report to the Pasteur Institute (4 August 1894) he described how he extracted soil from one house from eight to ten centimetres deep, and upon creating a culture ‘was surprised to find in my tubes colonies of the microbe of absolutely the same aspect as plague bacillus colonies’.^42^ Alas, being inoculated with one of the cultures, test-animals failed to die. Yersin was nonetheless happy with his findings, claiming that the inability of the culture to kill the test-animals was to be attributed to the attenuated state of the particular bacterial specimen.^43^ In the following days he repeated the experiment with soil from other infected houses, taking as a control sample soil from non-infected homes. Whilst not finding the bacillus in the latter, he once again discovered it in four out of ten of the infected houses. Following this, Yersin also investigated houses in Canton, which had also come under the bane of plague in the preceding months. This was of particular interest for, unlike Hong Kong, these had not been previously disinfected. There he also found the bacillus, attained as far as twenty to thirty centimetres deep, with no traces of it deeper than one metre. Yersin concluded: ‘The experiments permit [us] to 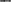 conclude that the microbe of plague exists in the soil where it probably undergoes a long evolution through which from being non-virulent it ends up becoming virulent again’.^44^ In his response to the Colonial Surgeon, Yersin claimed he experienced no difficulty in finding in the soil of infected houses ‘a little bacillus identical with regard to aspect and the culture of the plague bacillus’.^45^ Commenting on the fact that animals inoculated with the soil bacillus did not succumb to the disease, he remarked that the lack of virulence did not surprise him as he had previously found plague bacilli of equally low virulence in buboes of patients: ‘I have authentic cultivations of plague, which kill neither the *cobaye* [guinea pig] or the mouse, like the bacillus I have found in the soil’.^46^

Caught between two learned opinions, Lowson reported that he consulted Kitasato’s assistant, Dr Takaki, who was persuaded to conduct more soil experiments. What Takaki found was an organism that looked identical, but was reportedly different to the plague bacillus; a somewhat loosely defined verdict later authenticated by Kitasato, to whom soil specimens ‘from the worst houses’ were sent. This, in the eyes of Lowson, brought the matter to a close.^47^ In a particularly vitriolic letter to Kitasato dated 20 August 1894 he wrote:

Mein Blutreich Professor, I salute you, and hope that you will be able to prepare a new shell filled with pest bacilli for the damned Chinaman. If you can at the same time kill a man called Yersin, for God’s sake do so. He has led us a dance in a way but Takaki will tell you we have got the better of him. I now say that the bacilli are not formed deep in the mud – not deeper than half an inch. The question of digging up Taipingshan does not depend so much on account of the *Bacillus Kitasatoiensis* as on account of its general dirty condition.^48^

In the meantime, the Housing Committee had already moved unanimously to recommend the resumption of a large area (ten acres) in Taipingshan, and the demolition ‘preferably by fire’ of 384 houses.^49^ As shown by Chu, the decision would lead to a long exchange of appeals, objections and deliberation until the final enactment of the Taipingshan Resumption Order.^50^ What is less noted is that the Housing Committee also passed a verdict on the relation between soil and plague:

The Committee advise that the soil, after the demolition of the houses by fire, and after being disinfected, be removed to a depth of at least one foot, and that the surface, when again built upon, be covered with impervious material of approved thickness, any house drains discovered during these operations being entirely removed.^51^

As for Yersin, he faced the conclusion of the Housing Commission by summarily correcting his previous ‘mis-statement’, clarifying that plague is to be found two inches below the ground, and not eighteen inches as previously stated.^52^

## 2 The Soil as an Epistemic Thing

Establishing the soil as a medically legitimate and administratively pertinent aspect of plague research and intervention, the Hong Kong debate consolidated an entanglement between it and what historians have more broadly described as the ‘spatial formation and meaning’ of modern plague.^53^ Yet at the same time, this process also signalled an epistemological suspension. Rather than simply redefining the soil as an object of bacteriological experimentation, its examination under Kitasato’s and Yersin’s microscopes, and the divergent results and conclusions drawn from it, indicated that the discrepancy between the identification of the plague bacillus by the two scientists was not simply the result of haste or ambition, but (*contra* Latour and Cunningham) evidence of the laboratory’s inability to decide the big questions about plague.^54^ Positioned between the lab and the street, but also between conflicting colonial urban planning and public health agendas, real estate interests and epistemologies of plague, the question of the soil helped configure plague as a phenomenon unfolding, to paraphrase Shawn Michelle Smith, at the edge of medical sight.^55^ In short, the soil was taken for the locus where plague concealed itself both in terms of inter-epidemic latency and of it being elusive vis-à-vis the methods employed to ascertain it.

By August 1894 the soil had already assumed a significant position in what, following Hans-Jörg Rheinberger, we may call plague-related experimental systems that arose from the ontological fixing of the disease’s causative agent. However, in this process, the soil did not become ‘sufficiently stabilised’ to become ‘the technical repertoire’ of bacteriological and sanitarian ‘experimental arrangement(s)’.^56^ Instead, through its iterant difference, it was rendered, quite literally, the locus of epistemic and biopolitical unsettlement between different approaches to plague and its relation to urban forms and patterns.^57^ As a result, the soil was transformed into an epistemic thing that operated in the field of what Rheinberger, following Robert Merton, has called ‘unspecified ignorance’.^58^ *Not precisely knowing what one did not know* about the soil and its connection to plague became both a significant driver of scientific research and, as the case of India makes particularly clear, a fertile field for applied experiments in containing the disease through the manipulation of the urban environment.

## 3 True Recrudescence

The outbreak of plague in India in 1896 did not automatically lead to the resurfacing of soil as a suspect for harbouring or spreading of the disease. James Lowson, who was dispatched to plague-stricken Bombay, was quick to rehearse his rejection of any significant plague-soil connection: ‘One reason that so much stress has been laid on the question of infection from the soil’, he argued, ‘is that in former days it was stated that people living on the ground floor were usually infected first’.^59^ This, according to Lowson, was nothing but a truism, given that houses ‘in Oriental countries’ only have one floor – that being on the ground level. Allowing for the existence of more levels, he retorted, ‘cases have occurred on any and every floor’.^60^ Yet this opinion was to find a formidable opponent: James Cantlie, resident doctor in Hong Kong since 1888, mentor of Dr Sun Yat-sen, and co-founder of what would eventually become Hong Kong University.

One of the few British colonial doctors to befriend Yersin during his plague investigations, the Scottish physician had expressed in an article in the *British Medical Journal* (August 1894) the opinion that the disease was ‘miasmatic contagious’.^61^ Rather than being a survival of what is conventionally seen as miasmatic theory in an age of bacteriology, this was a complex aetiological model that would be, at least partially, adopted by other leading plague scientists, like Ernest Hanbury Hankin.^62^ Miasma, in the sense used by Cantlie, referred to the method rather than the agent of infection. For whilst accepting the bacteriological identity of the disease, he was also convinced that the notion that plague sprang from the earth was sound: ‘The dominant idea before even the bacteriologist or the microbiologist studied the matter was that the infection arose from the soil and there seem no facts to go upon to reject this creed’.^63^ This aetiological reasoning would be fully developed in a widely publicised lecture on the spread of plague, delivered before the Epidemiological Society of London on 18 December 1896:

What has been termed the ‘miasmatic method’ of infection bears distinctly upon the maintenance and epidemicity of plague. Miasmatic infection implies a soil-bred disease – a disease existing, as we assume malaria does, in the earth itself, in the water, or in some particular form of fermenting or decomposing material in which the germ finds a nidus. In a neighbourhood so infected the disease is endemic, as manifested by the fact that again and again the disease recurs – that after a few months of seemingly complete disappearance the disease crops up and runs a more or less severe course.^64^

What was the reason, he queried, that the disease was not apparent during the said interval? Was it because it was hibernating or because of variations in climatic conditions? For Cantlie the connection of plague with the soil was not simply a ‘creed’, but primarily a question: what was the precise relation between the ‘infection of the soil’ and human infection? Was the ‘soil bacillus’ identical to the human one? What was its virulence, and was it fixed or could it fluctuate? Equally important, and key to configuring plague’s virulence, was the question of whether certain environmental conditions were conducive to plague gaining or losing in virulence. This was a complex problem, as it brought into relation infrastructural, behavioural and climatological parameters. As regards the latter, the absence of rain was widely believed to foster plague in terms of dirt accumulating in people’s houses and bodies (it was often mentioned that the disease first struck Canton and Hong Kong after a severe spell of drought). Yet this was an oxymoron, as the lack of rain led to a dry soil, which was believed to be detrimental to plague, as ‘the bacterium is dependent on a sufficiency of moisture’.^65^ Still, whether they saw rain or draught as a catalyst of infection, few at the time would disagree with Cantlie’s opinion, that when climatic conditions necessary for the growth of the bacillus assumed the necessary quantity or quality then, ‘the revival of its infective power is readily manifested’.^66^ This was, in summary, a model that assumed plague to be a soil-bred bacterial disease, whose bacilli are retained in the environment and may, according to climatic conditions, or to other yet unknown reasons, attain different degrees of infective power or virulence.

For Cantlie the idea of soil infection raised the question of the medium of the disease. Using a parasitological metaphor he explained: ‘The medium of the soil may be to the infection of animals as the medium of water bearing the carcase [sic] of the dead mosquito is to the filarial-infected human being. Not only may it serve as a mere vehicle, but as a host, in which the parasite passes through a stage of its evolution’.^67^ This was a notion that clearly reflected Yersin’s hypothesis, expressed in his July 1894 response to Ayres: ‘It is possible that, in order to renew its virulence, [the bacillus] might have to make a long evolution in the earth’.^68^

According to this view, under certain conditions the soil functioned as the context of the development or transformation of the bacterium. In other words, the soil was considered as far more than an idle container of plague – it was its medium proper, in the sense that it was seen as giving rise to virulent forms of the pathogen after periods of dormancy or attenuation, such as described by Yersin. This idea of so-called true recrudescence had the potential of explaining the apparent seasonal periodicity of plague, but also why the disease disappeared and reappeared in relatively isolated areas, such as the Punjab. The term, adopted by the Indian Plague Commission, was linked to the prevailing notion that such phenomena were not primarily due to re-importation, but ‘to the infective material lying dormant for prolonged periods in the soil or in the houses’.^69^ This framed the soil not simply as one amongst many carriers of plague, but as the medium where the pathogen acquires its virulence, and where it can return and persevere during shorter or longer periods of latency, rendering itself invisible to medical authorities. A ground where colonial concerns about urban structures, native bodies and ways of life could merge and contend, the soil hence operated as a locus of materialisation as regards the invisibility of plague. It allowed, in other words, for the true character of the disease to be perceived as being-invisible, or concealed, with its epidemic or visible manifestation being only a temporary and perhaps misleading appearance. Being, as it were, at the edge of medical sight, was hence rendered not a technical limitation, but a pivot of the nature of plague.

## 4 Burning the ‘Food’ of Infection

A set of evidence that led many doctors on the ground to conclude that there was in fact a connection between the soil and plague was that, in the course of the first wave of the epidemic in India, no new plague cases developed in hospitals, where the soil was assumed to be thoroughly decontaminated.^70^ Though this formed a widely shared opinion, what remained debatable was exactly how the soil became contaminated, and how, in turn, the disease spread from it to humans or animals. Could it be, as Major Evans suggested, that patients contaminated the soil by passing on infected matter before they died? And could plague-infected soil spread the disease as wind, rain, animals or vehicles carried it afield, so as to ‘form new centres of infection in congenial soils’?^71^

Though no consensus was reached over such matters, a shared conviction amongst British colonial doctors in Hong Kong and British India was that plague could be contracted from the soil by walking barefoot or by sleeping on it, through wounds or breaks in the skin.^72^ While in the case of Hong Kong, the population targeted as engaging in this infection-prone habit were the so-called coolies, in Indian cities, like Calcutta, the blame fell on women. Stating that ‘plague is generally regarded as a so-called house disease, and one in which the soil contains and conveys the infection’; Frank Clemow, a leading authority on the disease at the time, argued that women in India were more probable to be infected, as ‘one of their most constant occupations appears to be the cleansing of brass and other vessels with earth scraped from the floor of their huts or from the road outside’.^73^ Yet rather than the doctrine of the infective soil simply leading to the colonial propagation of wearing shoes or using elevated beds, some of the most invasive and destructive anti-plague measures applied against plague during the first epidemic of the disease in India were linked to the notion that the earthen walls and floor of houses were catalysts of the infection.

Methods employed to counter the infective propensities of the soil, included pulling entire houses down, so as ‘to expose the soil to the air in case it did contain infection’, but also burning houses down, as well as first burning infected houses, then upturning the soil, and finally putting thorns on top of the site to prevent people from walking on it.^74^ In fact, one of the most drastic measures of this kind on record was not taken by the British, but by the Nizam of Hyderabad. Basing his decision on British advice that plague was a soil disease best exterminated by fire, the Nizam ordered the floors of infected houses, in both urban and rural settings, to be covered in burning cow-dung and kerosene oil. When that proved impractical, an even more elaborate method was employed. After floors were whitewashed to protect anti-plague staff from ‘infected dust’, they were destroyed by being dug-up and subjected to Deputy Plague Commissioner Stevens’ so-called kiln method.^75^ The method involved four steps: the removal of residents to temporary lodgings; the removal of all items from the house; the whitewashing of the floor and walls of the house, with the object of ‘ensur[ing] that every particle of the whitened surface is removed for burning, any white patch immediately showing an unremoved portion’; and finally, the construction and operation of the kiln:

A piece of ground, say 5 feet in diameter is converted over with two layers of ‘ooplies’ (dried cakes of cowdung). The earth from the floor of the infected house is removed to a depth of  inch, filled into tins or iron baskets, and then sprinkled over the ‘ooplies’ for a depth of  inch. In this a second layer of ‘ooplies’ to the depth of 2 inches is laid and again a layer of infected earth. A third or fourth layer, or as many as necessary, are superimposed in alternate layers of ‘ooplies’ and earth until a heap of the height of  to 2 feet is attained. Lastly the kiln is covered in from top to bottom with two layers of ‘ooplies’ and the kiln lighted.^76^

Leaving the kiln to burn for forty-eight hours was believed to get rid of plague: ‘All that now has to be done to the house is to fill in the floor with fresh field earth, which is subsequently moistened, rammed firm, and mud plastered’. With an added splash of perchloride of mercury solution on the new floor and walls, the house ‘is fit for occupation’.^77^

The non-reappearance of plague in the previously infected Hyderabad State villages was attributed to this method of ‘disinfection by burning in kiln’.^78^ In his examination by the Plague Commission (19 December 1898), Lieutenant Colonel Lawrie, the acting Plague Commissioner for the Hyderabad Territory, would defend the kiln method on account of its low cost, its popularity amongst the local populace and its unique ability to destroy not only ‘infection’, but also ‘the food that the plague microbe likes, and by which it is kept alive and toxic’.^79^ This idea was fostered by experiments showing an inoculated rat dying and an identical microbe to the one isolated in the dust being found in it in abundance.^80^ On the other hand, ‘when a mud floor is reduced to ashes by the kiln process of burning no trace of the microbe can be discovered in the ashes’.^81^

What appeared to be an innovative way of disinfecting the soil from plague bacteria, which it was supposedly harbouring, was not, however, destined to become a generally applied epidemic control method in India during its long struggle against the disease. In the following decades, the question of the soil as a container of plague or a medium through which the disease acquired true recrudescence was displaced by the new protagonists of plague research: the rat and its flea. The slow establishment and consolidation of plague as a zoonotic disease paralleled the demise of the soil from modern epidemiological reasoning. Up until the end of the nineteenth century, the great soil debates in Hong Kong and India still reverberated across the globe: from experiments in plague-stricken Taiwan (1899) and soil-removal operations in New South Wales (1900), to the great Honolulu Chinatown fire of 1899, the question of the soil continued to cast its shadow on the relation between plague and urban space.^82^ Still, while as late as 1905 we can still see the impact of these debates in instances like the official endorsement of the employment of a desiccating stove used to disinfect earthen floors from plague in the Punjab, the question of the soil would, in general, recede into a marginal, specialised topic of plague research, whose importance for epidemic control and prevention was conclusively diminished.^83^

## 5 Conclusion

What the question of the soil forged during the first half-decade of the third plague pandemic was not simply a bridge between miasmatic and germ theories of infection, but more importantly an epidemiological framework through which infection and recrudescence, as well as urban lifestyles and infrastructures could be understood in an integrated and materially tangible manner. More an epidemiological principle than just an epidemic source, the soil encompassed sanitary anxieties about working-class and colonial urban forms, bacteriological concerns with inoculation and dormancy, as well as native understandings of plague.

This paper has chosen not to approach the question of the soil as a moribund scientific controversy – or contradiction, in Rheinberger’s use of the term – that failed to lead to an ‘eventual solution’, contributing to the identity of plague.^84^ Instead, the aporetic nature of the soil as an epistemic thing has been approached as catalytic to questions regarding urban infrastructures and lifestyles and the role of their entanglement in the spread and persistence of plague in given localities. By operating within both bacteriological and sanitarian experimental systems – the microscope and fire – as ‘spaces of emergence’ of plague into a knowable and actionable category, the soil became the locus of the materialisation of the disease’s invisibility as a constitutive part of its urban inception and iteration.^85^ If the rat and its flea eventually came to assume the prominent position as ‘difference machines’ of plague research and intervention, one can but wonder if this would have been achieved without the initial problematisation of the soil as the breeding ground of plague.

## Footnotes

1.
Mauritius, Report of the Special Committee of the Council of Government on Plague, 16 May 1899, enclosed in Plague General Despatches 1900 January–May. Colonial Office, Series MH 19/259, CO 24828.

2.

David S. Barnes , The Great Stink of Paris and the Nineteenth-Century Struggle against Filth and Germs (Baltimore, MD: Johns Hopkins University Press, 2006).

3.
It should be noted that the bacteriological implication of the soil as a carrier or source of plague originally preceded and then run parallel to similar problematisations of typhoid fever in England, without, however, any direct reference on the part of colonial plague experts to typhoid-related research back in Britain; Jacob Steere-Williams, ‘Performing State Medicine during its “Frustrating” Years: Epidemiology and Bacteriology at the Local Government Board, 1870–1900’, *Social History of Medicine*, 28, 1 (2015), 82–107.

4.
Recent studies suggest evidence of plague remaining alive and virulent in the soil (for up to 40 weeks, indicated in the case of Ayyadurai *et al.* 2008); S. Ayyadurai, L. Houhamdi, H. Lepidi, C. Nappez, D. Raoult and M. Drancourt, ‘Long-Term Persistence of Virulent *Yersinia pestis*in Soil’, *Microbiology*, 154 (2008), 2865–71; R. J. Eisen, J. M. Petersen, C. L. Higgins, D. Wong, C. E. Levy, P. S. Mead, M. E. Schriefen, K. S. Griffith, K. L. Cage and C. B. Beard, ‘Persistence of *Yersinia pestis* in Soil under Natural Conditions’, *Emerging Infectious Diseases*, 14, 6 (June 2008), 941–3.

5.

H. H. Mollaret , ‘Conservation expérimentale de la peste dans le sol’, *Bulletin de la Société de pathologie exotique*, 56, 6 (November 1963), 1168–82.14156818

6.

*Ibid.*, 10. Mollaret conducted her own experiments with plague in the soil, acquiring positive results.

7.
House of Commons Parliamentary Papers, Session: 1894 [C.7461] [C.7545]. Hong Kong. Correspondence Relative to the Outbreak of Bubonic Plague at Hong Kong.

8.

Mary P. Sutphen , ‘Not What, but Where: Bubonic Plague and the Reception of Germ Theories in Hong Kong and Calcutta’, *Journal of History of Medicine and Allied Sciences*, 52 (January 1997), 81–113.10.1093/jhmas/52.1.819071848

9.

Shibasaburō Kitasato , ‘The Bacillus of Bubonic Plague’, *The Lancet*, 144, 3704 (25 August 1894), 428–30; Alexandre Yersin, ‘La peste bubonique á Hong Kong’, *Annales de l’ Institut Pasteur*, 8 (1894), 662–7.

10.

James A. Lowson , Handwritten Report, 16 May 1894, enclosed in Robinson to Ripon, 17 May 1894. Colonial Office. Original Correspondence: Hong Kong 1841–1951; Series 129/263, CO 10928.

11.

*Ibid*.

12.
House of Commons Parliamentary Papers, Session: 1878–9 [C. 2262], Plague. Papers Relating to the Modern History and Recent Progress of Levantine Plague; prepared from time to time by direction of the president of the Local Government Board, with other papers, 28. See also, C. Zuber, ‘Rapport sur une missione médicale en Russie: La peste du gouvernement d’Astrakhan’, *Recueil des travaux du Comité Consultatif d’hygiène publique de France et des actes officiels de l’administration sanitaire* (Paris: A. Lahure, 1880) vol. 9, 87–167.

13.

Carol A. Benedict , Bubonic Plague in Nineteenth-Century China (Stanford, CA: Stanford University Press, 1996), 107.10.1177/00977004880140020111620272

14.

Emile Rocher , La province Chinoise du Yunnan (Paris: Lerous, 1879); A. Rennie, ‘Report on the Plague Prevailing in Canton during the Spring and Summer of 1894’, *Imperial Customs Maritime Report for the Year Ended 30th September*, 1894, 47th and 48th issues; J. L. Michoud ‘Report on the Health of Mengtsz for the Year Ended 30th April 1894’, *China Imperial Maritime Customs Medical Reports*, 1894, Special Series, No. 2. Yersin also mentioned Rocher on the infection of rats and other animals in his original plague discovery article (*op. cit*, note 13). A key proponent of the theory that plague first strikes shorter and then taller animals was James Lowson; *Hong Kong Government Gazette*, GA 1895 no. 146; Medical Report on the Epidemic of Bubonic Plague in 1894 (incorporating J. A. Lowson, ‘The Epidemic of Bubonic Plague in Hong Kong, 1894’) (13 April 1895), 369–422.

15.
Enclosure ‘Gazette June 29, 1894’, in Robinson to Ripon 30 July 1894. Colonial Office. Original Correspondence: Hong Kong 1841–1951; Series 129/263, CO 13259.

16.
Barnes, *op. cit* (note 2); E. Cockayne, *Hubbub: Filth, Noise and Stench in England* (New Haven, CT: Yale University Press, 2007); M. Bradley (ed.) *Pollution and Propriety: Dirt and Hygiene in the Eternal City from Antiquity to Modernity* (Cambridge: Cambridge University Press, 2012). On the ‘occult nature of Victorian dirt’, see C. Herbert, ‘Rat Worship in Mayhew’s London’, *Representations,* 23 (Summer 1988), 1–24: 8.

17.
In R. Peckham, ‘Hong Kong Junk: Plague and the Economy of Chinese Things’, *Bulletin of the History of Medicine*, 90 (2016), 32–60: 44.10.1353/bhm.2016.001127040025

18.
See in particular D. S. Barnes ‘Cargo, “Infection”, and the Logic of Quarantine in the Nineteenth Century’, *Bulletin of the History of Medicine*, 88, 1 (Spring 2014), 75–101; P. Stallybrass and A. White, ‘The city: the sewer, the gaze and the contaminating touch’, in J. Farquhar and M. Lock (eds), *Beyond the Body Proper: Reading the Anthropology of Material Life* (Durham, NC: Duke University Press, 2007), 266–85; R. el-Khoury, ‘Polish and deodorize: paving the city in late eighteenth-century France’, in J. Drobnick (ed.) *The Smell Culture Reader* (Oxford: Berg, 2006), 18–28.10.1353/bhm.2014.001824769803

19.
Peckham, *op. cit.* (note 17).

20.

J.-F. Tsai , Hong Kong in Chinese History: Community and Social Unrest in the British Colony, 1842–1913 (New York: Columbia University Press, 1993). On plague and class in Hong Kong, see also M. P. Sutphen, ‘Rumoured power: Hong Kong, 1894 and Cape Town, 1901’, in Andrew Cunningham and Bridie Andrews (eds) *Western Medicine as Contested Knowledge* (Manchester: Manchester University Press, 1997), 241–61.

21.
Anon., ‘Plague in the Far East’, *British Medical Journal*, 2, 1860 (22 August 1896), 460.PMC251035620756399

22.

*Aoyoma’s Report upon the Bubonic Plague* (the substance of a report made to the Journal Club of the Johns Hopkins Hospital by Simon Flexner M.D.); offprint of the *Johns Hopkins Hospital Bulletin*, 66–7 (September–October 1896), 3.

23.

Cecilia Chu , ‘Combating nuisance: sanitation, regulation, and the politics of property in colonial Hong Kong’, in Robert Peckham and David M. Pomfret (eds), Imperial Contagions: Medicine, Hygiene and Cultures of Planning in Asia (Hong Kong: Hong Kong University Press, 2013), 17–36: 31; ‘Mr. Chadwick’s Reports on the Sanitary Condition of Hong Kong; With Appendices and Plans.’ Colonial Office, Eastern No. 38, CO 882/4/15.

24.
Correspondence re Improvement of Tai-Ping Shan, enclosed in Robinson to Ripon, 30 August 1894. Colonial Office. Original Correspondence: Hong Kong 1841–1951; Series 129/263, CO 17303.

25.

*Ibid*.

26.
Correspondence re Improvement of Tai-Ping Shan, *op. cit*. (note 24).

27.

*Ibid*.

28.

*Ibid*. ‘Resumption’ here referred to the repossession of the land and its re-development.

29.
Sutphen, *op. cit*. (note 8), 98.

30.
Correspondence re Improvement of Tai-Ping Shan, *op. cit*. (note 24).

31.

*Ibid*.

32.
Older miasmatic theories of plague relied on ‘the action of the heat in destroying plague’ as a proof of the disease not being contagious; Ph. Jenks, *An Essay on the Analogy of the Asiatic and African Plague and the American Yellow* (Philadelphia, PA: Hugh Maxwell, 1804), 14.

33.

*Hong Kong Government Gazette*, GA 1895 no. 117; Report, Scheme for the Improvement of the Resumed Area in Taipingshan (incorporating Correspondence No. 132 by Francis A. Cooper, Director of Public Works, 22 March 1895) (30 March 1895), 262–5: 264. Cooper retorted that it was sanitary measures and not the fire that eradicated plague in London. Even if fire were applied, Cooper reasoned, the heat would fail to penetrate the surface of the ground to a desirable effect.

34.
Correspondence re Improvement of Tai-Ping Shan, *op. cit*. (note 24).

35.
Chu, *op. cit*. (note. 23).

36.
Sutphen, *op. cit*. (note 8), 96–7. The Koch connection relates to the famous discovery of anthrax spores by the German doctor.

37.
On Lowson see T. Solomon, ‘Hong Kong, 1894: The Role of James A. Lowson in the Controversial Discovery of the Plague Bacillus’, *The Lancet*, 350, 9070 (5 July 1997), 59–62.10.1016/S0140-6736(97)01438-49217728

38.

*Hong Kong Government Gazette*, GA 1895 no. 146, *op. cit*. (note 14).

39.

*Ibid.*, 400.

40.
Correspondence re Improvement of Tai-Ping Shan, *op. cit.* (note 24).

41.

J. Andrew Mendelsohn , ‘The Microscopist of Modern Life’, *Osiris*, 2nd ser., 18, Science and the City (2003), 150–70.10.1086/64938212964593

42.
Archives Institut Pasteur, Fonds: Yersin Alexandre, Cote: YER.5 – Lieu : A1/13, my translation.

43.
Yersin appears enthusiastic about this in his correspondence to his mother; Archives Institut Pasteur, Fonds: Yersin Alexandre Cote: YER.Cor1 – Lieu: A1/13.

44.
Archives Institut Pasteur, Fonds: Yersin Alexandre Cote: YER.5 – Lieu: A1/13.

45.
Correspondence re Improvement of Tai-Ping Shan, *op. cit*. (note 24).

46.

*Ibid*.

47.

*Hong Kong Government Gazette*GA 1895 no. 146, *op. cit*. (note 14), 400.

48.
James A. Lowson to Kitasato Shibasaburo, Correspondence 10 August 1894, uncatalogued item at Kitasato Memorial Museum, Tokyo. Not in use at the time, the term *Bacillus Kitasatoiensis*was here clearly employed to establish Kitasato’s priority in the discovery of the bacillus which was generally referred to as *Pasteurella pestis* until 1944 when it was renamed *Yersinia pestis*, after Alexandre Yersin. On Lowson’s antipathy for Yersin and the wider Kitasato-Yersin conflict, see D. J. Bibel and T. E. Chen, ‘Diagnosis of Plague: An Analysis of the Yersin-Kitasato Controversy’, *Bacteriological Reviews*, 40, 3 (September 1976), 633–51, and Solomon, ‘Hong Kong, 1894’, *op. cit.* (note 37).10.1128/br.40.3.633-651.1976PMC41397410879

49.
Correspondence re Improvement of Tai-Ping Shan, *op. cit*. (note 24).

50.
Chu, *op. cit*. (note 23).

51.
Correspondence re Improvement of Tai-Ping Shan, *op. cit*. (note 24).

52.
Yersin gives no explanation as to how this correction was reached; *Hong Kong Government Gazette*, GA 1895 no. 117; Report, Scheme for the Improvement of the Resumed Area in Taipingshan (incorporating Correspondence No. 132 by Francis A. Cooper, Director of Public Works, 22 March 1895) (30 March 1895), 262–5.

53.

Susan Craddock , City of Plagues: Disease, Poverty, and Deviance in San Francisco (Minneapolis, MN: University of Minnesota Press, 2004) , 8.16841409

54.

Bruno Latour , The Pasteurization of France (Cambridge, MA: Harvard University Press, 1993). Andrew Cunningham, ‘Transforming plague, the laboratory and the identity of infectious disease’, in A. Cunningham and P. Williams (eds), *The Laboratory Revolution in Medicine* (Cambridge: Cambridge University Press, 1992), 209–44. On the question of the limitations of the laboratory’s impact on plague research, see Christos Lynteris, *Ethnographic Plague: Configuring Disease on the Chinese-Russian Frontier* (London: Palgrave Macmillan, 2016).

55.

Shawn Michelle Smith , At the Edge of Sight: Photography and the Unseen (Durham, NC: Duke University Press, 2013).

56.

Hans-Jörg Rheinberger , Toward a History of Epistemic Things: Synthesizing Proteins in the Test Tube (Stanford, CA: Stanford University Press, 1997), 29.

57.
On the notion of difference in experimental systems see, *ibid*, and Hans-Jörg Rheinberger, ‘Difference Machines: Time in Experimental Systems’, *Configurations,*23, 2 (Spring 2015), 165–76.

58.

Hans Blumenberg , *Theorie der Unbegrifflichkeit* [*The Theory of Unconceptuality*] (Frankfurt am Main: Suhrkamp, 2007); Rheinberger, ‘Difference Machines’, *op. cit.* (note 57).

59.

James A. Lowson , Report on the Epidemic of Plague [in Bombay] from 22 February to 16 July 1897. British Library: Asian and African Studies; IOR/V/27/856/13.

60.

*Ibid*.

61.

James Cantlie , ‘The Plague in Hong Kong’, *The British Medical Journal*, 2, 1756 (25 August 1894), 423–7.

62.
Hankin employed exactly the same term in his influential 1905 paper, ‘On the Epidemiology of Plague’, *The Journal of Hygiene*, 5, 1 (January 1905), 48–83. As Mollaret (*op. cit*. (note 5)) has argued, Hankin’s actual approach to the soil appears to be self-contradictory; though all his experiments in procuring a plague culture from the soil proved fruitless, he nonetheless claimed that the transfer of the bacterium from the rat’s body into the soil might be necessary in order for it to retain its virulence across time.

63.

James Cantlie , ‘The Spread of Plague’, *The Lancet*, 1, 3828 (9 January 1897), 89.

64.

*Ibid*, 85. The lecture would be printed in two instalments in *The Lancet*as well as in pamphlet form.

65.

*Ibid*, 89.

66.

*Ibid*, 85.

67.

*Ibid*, 89.

68.
Correspondence re Improvement of Tai-Ping Shan, *op. cit*. (note 24).

69.
House of Commons Parliamentary Papers, Session: 1900 [Cd.810]; Indian Plague Commission, 1898–9. Report of the Indian Plague Commission with appendices and summary. Vol. V, 176.

70.
House of Commons Parliamentary Papers, Session: 1900 [Cd.140] Indian Plague Commission, 1898–9. Minutes of Evidence Taken by the Indian Plague Commission with appendices. Vol. II. Evidence taken from 11th January 1899 to 8th February 1899, 16,149; 1,947; 21,197; 21,537.

71.
House of Commons Parliamentary Papers, Session 1900 [Cd.139] Indian Plague Commission, 1898–9. Minutes of Evidence Taken by the Indian Plague Commission with appendices. Vol. I. Evidence taken from 29th November 1898 to 5th January 1899, 6,898–6,900. The porosity of the soil was another factor considered, following Pettenkofer (*Ibid*: 8,311–25).

72.
See in particular, House of Commons Parliamentary Papers, Session: 1900 [Cd.140], *op. cit.* (note 70), 15,198; House of Commons Parliamentary Papers, Session: 1900 [Cd.141] Indian Plague Commission, 1898–9. Minutes of Evidence Taken by the Indian Plague Commission with appendices. Vol. III, 17,775; *Hong Kong Government Gazette* GA 1895 no. 146, *op. cit.* (note 14), 375. This notion would be further promoted by comparative research conducted by Kitasato’s partner Dr Aoyoma in Japanese-ruled Taiwan; House of Commons Parliamentary Papers, Session: 1900 [Cd.810], *op. cit* (note 69). It should be noted here that the eventual discrediting of the soil hypothesis relied on evidence that there was no difference in inguinal bubo prevalence between ‘booted Australians’ and ‘the barefooted Indians of Bombay’ – ‘If the plague infection was derived from the soil the latter class of person ought to develop more groin buboes than the former’; W.B. Bannerman, ‘The Spread of Plague in India’, *The Journal of Hygiene*, 6, 2 (April 1906), 179–211: 209.

73.

Franck Clemow , ‘The Plague in Calcutta’, *The Lancet*, 2, 3916 (17 September 1898), 738–42: 741.

74.
House of Commons Parliamentary Papers, Session 1900 [Cd.139], *op. cit* (note 71), 1,097; House of Commons Parliamentary Papers, Session: 1900 [Cd. 140], *op. cit*. (note 70), 9,168.

75.
Anon., ‘The Plague in Hyderabad State: A Report by Lieutenant-Colonel Lawrie I.M.S.’, *The Lancet*, 1, 3935 (28 January 1899), 249–350: 250.

76.

A. H. Stevens , ‘Memorandum on the Treatment of Plague-Infected Premises and the Destruction of the Infection by the Kiln-Process of Burning’, enclosed in House of Commons Parliamentary Papers, Session 1900 [Cd.139], *op. cit*. (note 71).

77.

*Ibid*.

78.
Anon., ‘The Plague’, *British Medical Journal*2, 1982 (24 December 1898), 1906–07.

79.
House of Commons Parliamentary Papers, Session 1900 [Cd.139], *op. cit*. (note 71).

80.

*Ibid*.

81.

*Ibid*; see also, Anon., ‘The Plague in Hyderabad State’, *op. cit*. (note 75), 250.

82.
House of Commons Parliamentary Papers, Session 1901 [Cd.748]; Local Government Board. Reports and papers on bubonic plague, by Dr R. Bruce Low, 375–6.

83.
Punjab. Plague Department, Punjab Plague Hand-book, 1905. Lahore, 1905; British Library: Asian and African Studies; IOR/V/27/856/58.

84.
Rheinberger, ‘Difference Machines’, *op. cit.* (note 57), 166.

85.

*Ibid*., 168.

